# CMV infections after HSCT: prophylaxis and treatment

**DOI:** 10.1007/s44313-025-00081-7

**Published:** 2025-06-03

**Authors:** Haerim Chung

**Affiliations:** Division of Hematology, Department of Internal Medicine, Severance Hospital, Yonsei University College of Medicine, 50‑1 Yonsei‑Ro, Seodaemun‑Gu, Seoul, 03722 Republic of Korea

**Keywords:** Cytomegalovirus, Prophylaxis, Preemptive treatment

## Abstract

Cytomegalovirus (CMV) infection remains a major complication in recipients of hematopoietic stem cell transplantation (HSCT) and contributes significantly to morbidity and mortality. Effective CMV prevention and management are essential for improving transplant outcomes. Preventive strategies include antiviral prophylaxis and preemptive treatments (PET). Letermovir, a terminase complex inhibitor, has become the standard of care for primary prophylaxis in CMV-seropositive recipients because of its efficacy and favorable safety profile. PET involves regular monitoring of CMV DNAemia via polymerase chain reaction (PCR) and initiation of antiviral therapy, most commonly ganciclovir or valganciclovir, upon detection of early viral reactivation. Refractory or resistant CMV infections present a significant therapeutic challenge and often require switching to a different antiviral class while awaiting genotypic resistance testing. Maribavir, a UL97 kinase inhibitor, has demonstrated superior efficacy and improved tolerability compared to conventional therapies in the phase 3 SOLSTICE trial, making it a promising therapy for refractory or resistant CMV. Optimal CMV management requires a risk-adapted, individualized approach that integrates prophylaxis, early detection, and timely intervention to reduce CMV-related complications.

## Introduction

Cytomegalovirus (CMV) infection remains a major clinical challenge in patients undergoing hematopoietic stem cell transplantation (HSCT), highlighting the need for preventive and therapeutic strategies [1]. CMV, a member of the Herpesviridae family, is characterized by its ability to establish latency and reactivity under immunosuppressive conditions such as those following HSCT. [2] CMV reactivation can result in a spectrum of clinical manifestations ranging from asymptomatic viremia to severe end-organ diseases, including pneumonia, retinitis, and gastrointestinal complications. These outcomes can significantly affect patient prognosis and overall survival. Therefore, continuous monitoring and timely intervention are essential for mitigating CMV-related morbidity and mortality. A tailored approach to antiviral therapy combined with vigilant surveillance throughout the post-transplant period is critical for the effective management of CMV infections.

Patients undergoing allogeneic HSCT are at an increased risk of CMV infection owing to several contributing factors. One of the primary risk factors is the CMV serostatus of the donor and recipient, with mismatched serostatuses, such as a CMV-seropositive donor and a CMV seronegative recipient, significantly increasing the risk of CMV reactivation [3]. In the Korean population, the CMV seropositivity rate exceeds 90%, indicating that the majority of transplant recipients are CMV-seropositive, and are therefore, considered to be at a high risk for CMV infection following transplantation. The use of immunosuppressive agents, including anti-thymocyte globulin (ATG) and corticosteroids, impairs CMV-specific immunity [4]. Graft-versus-host disease (GVHD) is another major risk factor. Studies have shown a dose-dependent relationship between GVHD and CMV reactivation, with higher grades of acute GVHD associated with increased CMV replication. Notably, this relationship is bidirectional, as CMV reactivation may increase the risk of developing acute GVHD [5, 6]. In addition, human leukocyte antigen (HLA) mismatch between donor and recipient can contribute to immune dysregulation, further elevating the risk of CMV infection. Other factors, such as patient age and underlying diseases, may also influence susceptibility. Recognizing and managing these diverse risk factors are essential for the development of effective CMV prevention and treatment strategies for HSCT recipients.

### Prophylaxis

Two primary strategies are used to prevent CMV infection in HSCT recipients: prophylaxis and preemptive treatment (PET). Prophylaxis involves administration of antiviral medications to prevent CMV infection. This approach is recommended for high-risk patients. PET is based on regular monitoring of the CMV load, typically via polymerase chain reaction (PCR) assays, with treatment initiated upon the early detection of viral reactivation. This approach enables timely intervention before the development of clinical disease, thereby reducing the risk of CMV-related complications. In recent practice, a hybrid strategy combining a defined period of prophylaxis with PET is increasingly being adopted with the aim of balancing early prevention with reduced antiviral exposure over time [7].

Antiviral prophylactic strategies for the prevention of CMV reactivation and infection have advanced considerably, particularly in high-risk patients. Letermovir, approved by the U.S. Food and Drug Administration (FDA) in 2017, represents a significant milestone in CMV prevention. Unlike conventional DNA polymerase inhibitors, letermovir targets the viral terminase complex, specifically, the UL56 subunit, thereby inhibiting CMV replication through a novel mechanism of action [8]. In a pivotal phase 3 randomized controlled trial, letermovir prophylaxis significantly reduced the incidence of clinically significant CMV infection in allogeneic HSCT recipients compared with placebo (37.5% vs. 60.6%, *P* < 0.001) [9]. Its efficacy has also been demonstrated in high-risk populations, with recent studies indicating that extending prophylaxis to 200 days post-transplantation is both effective and well-tolerated, contributing to a reduction in late-onset CMV infections [10]. Letermovir has demonstrated a favorable safety profile with no significant adverse effects such as myelosuppression or nephrotoxicity. Importantly, among patients who initiated letermovir treatment before engraftment, the time to engraftment was comparable between the letermovir and placebo groups, supporting its safe use during the early post-transplantation period, including the pre-engraftment phase. In contrast to ganciclovir, which is associated with considerable myelosuppression and nephrotoxicity limiting its utility in prophylactic settings, [11] letermovir offers a safer alternative to CMV prophylaxis in HSCT recipients. Based on these clinical outcomes, letermovir prophylaxis has been approved for reimbursement in South Korea for use in CMV-seropositive HSCT recipients.

For primary CMV prophylaxis in CMV-seropositive allogeneic HCT recipients, letermovir is typically administered orally or intravenously (IV) at a dose of 480 mg/day (240 mg/day when co-administered with cyclosporine). Its availability as both oral and intravenous formulations allows for flexible administration. However, as letermovir lacks activity against other herpes viruses, concurrent administration of an anti-herpes agent such as acyclovir is recommended to provide comprehensive prophylactic coverage.

CMV blips or transient low-level CMV DNAemia may occur during letermovir prophylaxis owing to several underlying mechanisms. Letermovir inhibits the CMV terminase complex, which prevents the packaging and maturation of viral DNA into infectious virions. However, it does not inhibit the initial steps of viral DNA replication. As a result, non-infectious or incomplete viral particles may still be produced, and their DNA can be detected using sensitive PCR-based assays, even in the absence of true viral replication or disease [12]. These blips are typically self-limiting and do not progress to CMV disease. However, the authors highlight the importance of ongoing viral load monitoring during letermovir prophylaxis. Unless there is a sustained or increasing trend in viral DNA levels, treatment modifications are generally unnecessary.

### PET

PET strategies for managing CMV infections after HSCT focus on early detection and intervention to prevent CMV disease progression. This approach involves regular monitoring of the CMV viral load and the initiation of antiviral treatment when viremia is detected, with the aim of preventing progression to CMV end-organ disease [13]. The CMV viral load is typically monitored by PCR at least weekly for the first 100 days post-transplantation. The duration of monitoring may be extended for patients with a history of CMV infection, those with mismatched or unrelated donors, or those receiving glucocorticoids for GVHD.

The specific cutoff values for initiating PET in the management of CMV infections following allogeneic HSCT are not universally standardized and may vary across institutions and clinical guidelines. However, commonly used thresholds include a CMV viral load of approximately 500 IU/mL for high-risk patients and 1,000 IU/mL for low-risk patients. Despite the lack of consensus on the exact PCR-based threshold, treatment is generally initiated in high-risk patients when CMV DNA levels reach or exceed 500 IU/mL. Ultimately, the decision to initiate PET is guided by a combination of viral load, patient risk stratification, and institutional protocols [14].

Ganciclovir is the most commonly used agent for the PET of CMV infection and is typically administered at a dose of 5 mg/kg IV every 12 h. Valganciclovir, an oral prodrug of ganciclovir, offers equivalent efficacy at a dose of 900 mg twice daily and may reduce the need for hospitalization. However, both these agents are associated with a risk of myelosuppression. Foscarnet, administered at a dose of 60 mg/kg IV every 12 h, serves as an alternative in cases where myelotoxicity is a concern, although its use is limited by the potential for nephrotoxicity and electrolyte disturbances. The selection of antiviral therapy is influenced by factors such as the time elapsed since transplantation, patient’s risk of drug-related toxicity, and prior antiviral exposure. Notably, the prior use of letermovir prophylaxis does not affect the choice of PET agents. Treatment is generally continued for two weeks or until CMV PCR becomes negative, with the duration extended as needed in cases of persistent viremia.

PET offers a targeted approach to CMV management in transplant recipients, potentially minimizing unnecessary antiviral exposure while effectively preventing progression to CMV disease. However, the choice of the antiviral agent and treatment duration should be tailored to individual patient factors and clinical responses.

### Refractory or resistant CMV

Refractory CMV infection is defined as the failure to achieve virological control despite at least two weeks of appropriately administered antiviral therapy. This is typically characterized by a ≥ 1 log₁₀ IU/mL increase in CMV viral load or by persistent, fluctuating viremia with changes of < 1 log₁₀. Although refractory infections do not necessarily indicate antiviral resistance, they do indicate a suboptimal treatment response and warrant further evaluation. A subset of refractory cases is classified as resistant CMV infection, in which specific genetic mutations associated with antiviral resistance have been identified [15]. The most commonly implicated genes are UL97 and UL54. Mutations in UL97 confer resistance to ganciclovir and valganciclovir by disrupting viral kinases responsible for drug phosphorylation. UL54 gene mutations, which affect the viral DNA polymerase, may confer resistance to multiple antiviral agents, including ganciclovir, foscarnet, and cidofovir, depending on the mutation site [16].

When refractory or resistant CMV disease is suspected, immunosuppression should be reduced, if possible, and antiviral drugs should be switched to a different class while awaiting genotype resistance testing. Genotypic analysis should include sequencing of all relevant target genes using either Sanger or next-generation sequencing (NGS). Low-level variants detected by NGS require cautious interpretation and confirmation through repeat testing. If there is no virological improvement within two weeks of appropriate antiviral therapy, repeat genotyping should be performed for emerging resistance [17]. Traditionally, high-dose ganciclovir, foscarnet, and cidofovir have been used; however, they carry significant toxicity risks. Recently, maribavir has emerged as an effective option for refractory/resistant CMV, offering improved tolerability and fewer side effects than conventional agents.

A phase 3 clinical trial, the SOLSTICE study [18], was conducted to evaluate the efficacy and safety of maribavir in the treatment of refractory CMV infections, with or without documented resistance, in recipients of solid organ transplantation (SOT) and HSCT. In this randomized trial, patients received either maribavir 400 mg twice daily for eight weeks or investigator-assigned therapy (IAT), which included ganciclovir, valganciclovir, foscarnet, and cidofovir. At week 8, CMV viremia clearance was achieved in 55.7% of the patients in the maribavir group compared to 23.9% in the IAT group, demonstrating the superior efficacy of maribavir. Favorable outcomes with maribavir were consistent across pre-specified subgroups, including HSCT recipients, patients with baseline genotypic resistance to IAT, and those with refractory (non-resistant) CMV infection. Moreover, compared with conventional therapies, maribavir treatment was associated with a lower incidence of neutropenia and acute kidney injury. These findings establish maribavir as a promising alternative treatment for resistant and refractory CMV infections. Importantly, UL97 mutations conferring resistance to maribavir do not confer cross-resistance to ganciclovir, and vice versa, offering the potential for sequential therapy based on resistance profiles. However, maribavir demonstrates limited penetration across the blood–brain barrier and its use is not recommended in patients with CMV meningoencephalitis. The AURORA study evaluated maribavir as a positron emission tomography (PET) drug for treating CMV infection in HSCT recipients. Despite encouraging results from phase 2 trials, this phase 3 study did not meet its primary endpoint and showed no significant benefit over valganciclovir in preventing CMV infections [19]. As a result, maribavir cannot be recommended as a first-line option for PET. However, it demonstrated a more favorable safety profile than valganciclovir, with fewer adverse effects, such as myelosuppression. Therefore, maribavir may be considered in select cases in which conventional antivirals pose a high risk of toxicity.

Foscarnet is an alternative therapy for refractory CMV infections, particularly those involving the central nervous system or ocular tissue. Unlike ganciclovir, foscarnet directly inhibits viral DNA polymerase, rendering it effective against CMV strains harboring the UL97 or UL54 mutations. However, its use is limited by significant toxicity, especially nephrotoxicity, which necessitates close renal monitoring during treatment. Cidofovir, another antiviral agent, is a nucleotide analog primarily implicated in CMV retinitis. It also targets the viral DNA polymerase and is considered in cases of refractory CMV infection. Similar to foscarnet, its clinical use is constrained by the risk of nephrotoxicity, requiring careful monitoring of renal function. Emerging therapies, such as adoptive cellular immunotherapy and leflunomide, may offer additional benefits for refractory infections, although further research is needed to clarify their efficacy and clinical applicability.

## Conclusion

The effective management of CMV infection in HSCT recipients requires a comprehensive and individualized approach that includes risk assessment, prophylaxis, preemptive monitoring, and prompt treatment. Letermovir is the current standard for prophylaxis, whereas ganciclovir-based PET remains central to early intervention. In refractory or resistant cases, maribavir is a safer and more effective alternative, although its use for PET is limited. Continued research and development of novel therapies is critical for optimizing care and reducing CMV-related complications.

## Data Availability

No datasets were generated or analysed during the current study.
